# The Photoluminescent Properties of New Cationic Iridium(III) Complexes Using Different Anions and Their Applications in White Light-Emitting Diodes

**DOI:** 10.3390/ma8095296

**Published:** 2015-09-14

**Authors:** Hui Yang, Guoyun Meng, Yayun Zhou, Huaijun Tang, Jishou Zhao, Zhengliang Wang

**Affiliations:** Key Laboratory of Comprehensive Utilization of Mineral Resources in Ethnic Regions, Joint Research Centre for International Cross-border Ethnic Regions Biomass Clean Utilization in Yunnan, School of Chemistry & Environment, Yunnan Minzu University, Kunming 650500, China; E-Mails: yanghui0304@foxmail.com (H.Y.); mengguoyun@sina.com (G.M.); zhou-yayun@foxmail.com (Y.Z.); zhaojishou@163.com (J.Z.)

**Keywords:** cationic iridium(III) complex, photoluminescence, white light-emitting diode, blue GaN chip

## Abstract

Three cationic iridium(III) complexes [Ir(ppy)_2_(phen)][PF_6_] (C1), [Ir(ppy)_2_(phen)]_2_SiF_6_ (C2) and [Ir(ppy)_2_(phen)]_2_TiF_6_ (C3) (ppy: 2-phenylpyridine, phen: 1, 10-phenanthroline) using different anions were synthesized and characterized by ^1^H Nuclear magnetic resonance (^1^HNMR), mass spectra (MS), Fourier transform infrared (FTIR) spectra and element analysis (EA). After the ultraviolet visible (UV-vis) absorption spectra, photoluminescent (PL) properties and thermal properties of the complexes were investigated, complex C1 and C3 with good optical properties and high thermal stability were used in white light-emitting diodes (WLEDs) as luminescence conversion materials by incorporation with 460 nm-emitting blue GaN chips. The integrative performances of the WLEDs fabricated with complex C1 and C3 are better than those fabricated with the widely used yellow phosphor Y_3_Al_5_O_12_:Ce^3+^ (YAG). The color rendering indexes of the WLEDs with C1 and C3 are 82.0 and 82.6, the color temperatures of them are 5912 K and 3717 K, and the maximum power efficiencies of them are 10.61 Lm·W^−1^ and 11.41 Lm·W^−1^, respectively.

## 1. Introduction

More and more interest is focused on white light-emitting diodes (WLEDs), because of their high efficiency, long lifetime, energy-saving and environmentally friendly properties [1,2,3]. At present, the commercial WLEDs are mainly obtained by the combination of yellow phosphor Y_3_Al_5_O_12_:Ce^3+^ (YAG) with blue GaN-LED chips (λ_em_ ≈ 460 nm). It is well known that the main emission wavelength of the YAG is in the greenish yellow region [4]. Thus, WLEDs fabricated with YAG have low color rendering index (*R*_a_) and high color temperature (*T*_c_) because of the absence of red components in their spectra [5,6,7]. In order to enhance the emission of YAG in red regions, YAG is optimized by doping with some rare earth ions (such as Eu^3+^, or Pr^3+^) [6,7,8]. Although optimized YAG exhibit slightly red emission, but the yellow emission of the phosphors is obviously decreased. Hence, the development of new yellow phosphors for WLEDs based on blue LED chips is urgently needed. 

Recently, many organic luminescent conversion materials have also been used in LEDs, such as organic rare earth complexes [9,10,11], luminescent polymers [12,13,14] and small-molecule fluorescent dyes [15,16]. Cationic iridium(III) complexes with organic ligands composed of organic iridium(III) complex cation and inorganic acid anion (such as PF_6_^−^, ClO_4_^−^ and BF_4_^−^) have been widely applied in light-emitting electrochemical cells (LECs) [17,18,19] and organic light-emitting diodes (OLEDs) [20,21,22,23], as well as used as highly efficient luminescent materials in metal-oxide/metal-organic frameworks (MOFs) for LEDs and chemical sensors [24,25,26] because of their excellent photochemical and photophysical properties, such as high efficiency of 100% theoretical internal quantum efficiency, excellent color tenability via various ligands, short triplet state lifetimes, high thermal and photic stability and so on. These properties of cationic iridium(III) complexes meet the requirement of LEDs.

In this paper, three cationic iridium(III) complexes were synthesized with different anion sources, and their photoluminescence (PL) properties were investigated. Finally, the performances of WLEDs based on them were investigated. 

## 2. Experimental Section

### 2.1. Synthesis and Fabrication

**Figure 1:**
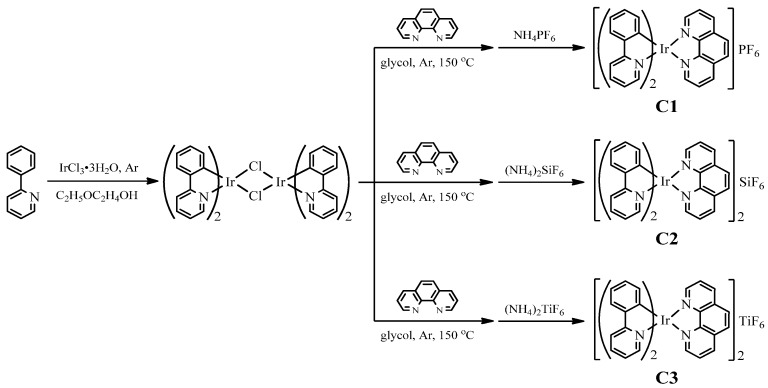
Synthetic route and chemical structures of the cationic iridium(III) complexes.

All reagents and chemicals are of analytical grade and used as supplied without further purification unless otherwise stated. The cationic iridium(III) complexes were synthesized according to our previous work [20,21,22], as shown in Figure 1. The chloro-bridged dimer (ppy)_2_Ir(μ-Cl)_2_Ir(ppy)_2_ (643 mg, 0.60 mmol, ppy:2-phenylpyridine) and 1,10-phenanthroline (phen, 237.6 mg, 1.2 mmol) were added into glycoland then kept at 150 °C in Ar atmosphere with stirring for 16 h. After being cooled to room temperature, 10 mL 0.3 mol·L^−1^ aqueous solution of ammonium salts NH_4_PF_6_, (NH_4_)_2_TiF_6_ or (NH_4_)_2_SiF_6_ was added with stirring, respectively. After the counter ion-exchange reaction from Cl^−^ to PF_6_^−^, TiF_6_^2−^ or SiF_6_^2−^ [27], plentiful floccules precipitate appeared. The precipitate was filtered, washed with water and dried in vacuum. The crude product was purified by column chromatography on silica gel with a mixture of CH_2_Cl_2_ and ethanol (volume ratio, 10:1) as eluent. All complexes were characterized by ^1^H Nuclear magnetic resonance (^1^HNMR), mass spectra (MS), elemental analysis (EA) and infrared spectra (IR). Yellow phosphor YAG was synthesized according to the reference [28]. The stoichiometric mixtures of Y_2_O_3_, Al(OH)_3_ and CeO_2_ were ground and fired at 1300 °C for 8 h in reducing atmosphere (N_2_:H_2_ = 95:5).

The series of WLEDs were fabricated by coating the mixture of epoxy resin and iridium(III) complexes or YAG phosphors on GaN chips.

### 2.2. Characterization

^1^HNMR spectra were recorded on a Bruker AV400 spectrometer operating at 400 MHz. Elemental analyses (EA) were performed on a Vario EL III Elemental Analysis Instrument. Mass spectra (MS) were obtained on a Bruker amaZon SL liquid chromatography mass spectrometer (LC-MS) with an electrospray ionization (ESI) interface using methanol as matrix solvent. Infrared spectra (IR) were recorded using a Fourier transform infrared spectrometer (IS10). Excitation and emission spectra were documented on a Cary Eclipse FL1011M003 (Varian, Palo Alto, CA, USA) spectrofluorometer, and the xenon lamp was used as excitation source. Thermogravimetric (TG) analysis was carried out up to 700 °C in N_2_ atmosphere with a heating speed of 10.0 K/min on a NETZSCH STA 449F3 thermogravimetric analyzer. The electroluminescent spectra of LEDs were recorded on a high-accuracy array spectrometer (HSP6000, HongPu Optoelectronics Technology Co. Ltd., Hangzhou, China). 

[Ir(ppy)_2_(phen)][PF_6_] (C1), yellow solid, yield: 85%, ^1^HNMR (400 MHz, CD_3_OD, 25 °C, ppm): 8.77 (d, 2H, ^3^*J* = 8.0 Hz, ArH), 8.36 (d, 2H, ^3^*J* = 4.8 Hz, ArH), 8.30 (s, 2H, ArH), 8.13 (d, 2H, ^3^*J* = 8.0 Hz, ArH), 7.86–7.93 (m, 4H, ArH), 7.80 (t, 2H, ^3^*J* = 8.4 Hz, ArH), 7.45 (d, 2H, ^3^*J* = 5.6 Hz, ArH), 7.08 (t, 2H, ^3^*J* = 7.6 Hz, ArH), 6.95 (t, 2H, ^3^*J* = 8.0 Hz, ArH), 6.90 (t, 2H, ^3^*J* = 7.6 Hz, ArH), 6.41 (d, 2H, ^3^*J* = 7.6 Hz, ArH). FTIR (KBr, cm^−1^): 3452, 3131, 1693, 1657, 1609, 1580, 1476, 1123, 1068, 964, 847, 617, 560, 539, 517. ESI-MS (*m*/*z*, being shown in Figure S1): 681.1 [M-PF_6_]^+^. Element Anal. Calc. For C_34_H_24_F_6_IrN_4_P(%): C, 49.45; H, 2.93; N, 6.78; Found(%): C, 49.32; H, 2.86; N, 6.60. 

[Ir(ppy)_2_(phen)]_2_SiF_6_ (C2), yellow solid, yield: 40%. ^1^HNMR (400 MHz, CDCl_3_, 25 °C, ppm): 8.93 (d, 4H, ^3^*J* = 8.0 Hz, ArH), 8.44 (s, 4H, ArH), 8.25 (d, 4H, ^3^*J* = 4.8 Hz, ArH), 7.88–7.93 (m, 8H, ArH), 7.72–7.73 (m, 8H, ArH), 7.31 (d, 4H, ^3^*J* = 5.6 Hz, ArH), 7.08 (t, 4H, ^3^*J* = 7.6 Hz, ArH), 6.97 (t, 4H, ^3^*J* = 7.6 Hz, ArH), 6.89 (t, 4H, ^3^*J* = 6.4 Hz, ArH), 6.40 (d, 4H, ^3^*J* = 7.6 Hz, ArH). FTIR (KBr, cm^−1^): 3453, 3133, 1694, 1657, 1461, 1390, 1350, 1264, 1123, 1068, 994, 954, 864, 821, 764, 618, 562, 539, 517. ESI-MS (*m*/*z*, being shown in Figure S1): 681.1 [1/2(M-SiF6)]^+^. Element Anal. Calc. For C_68_H_48_F_6_Ir_2_N_8_Si(%): C, 54.32; H, 3.22; N, 7.45; Found(%): C, 54.14; H, 3.53; N, 7.52. 

[Ir(ppy)_2_(phen)]_2_TiF_6_ (C3), yellow solid, yield: 73%. ^1^HNMR (400 MHz, CDCl_3_, 25 °C, ppm): 9.00 (d, 4H, ^3^*J* = 8.0 Hz, ArH), 8.49 (s, 4H, ArH), 8.25 (d, 4H, ^3^*J* = 4.8 Hz, ArH), 7.90–7.93 (m, 8H, ArH), 7.72–7.74 (m, 8H, ArH), 7.32 (d, 4H, ^3^*J* = 6.0 Hz, ArH), 7.09 (t, 4H,^3^*J* = 7.2 Hz, ArH), 6.98 (t, 4H, ^3^*J* = 8.0 Hz, ArH), 6.90 (t, 4H, ^3^*J* = 6.8 Hz, ArH), 6.40 (d, 4H, ^3^*J* = 7.6 Hz, ArH). FTIR (KBr, cm^−1^): 3451, 3131, 1694, 1657, 1606, 1460, 1381, 1347, 1265, 1124, 1069, 994, 955, 865, 823, 762, 616, 562, 539, 518. ESI-MS (*m*/*z*, being shown in Figure S1): 681.1 [1/2(M-TiF6)]^+^. Element Anal. Calc. For C_68_H_48_F_6_Ir_2_N_8_Ti(%): C, 53.61; H, 3.18; N, 7.36; Found(%): C, 53.85; H, 3.45; N, 7.62.

## 3. Results and Discussion

### 3.1. UV-Vis Absorption Spectra

**Figure 2:**
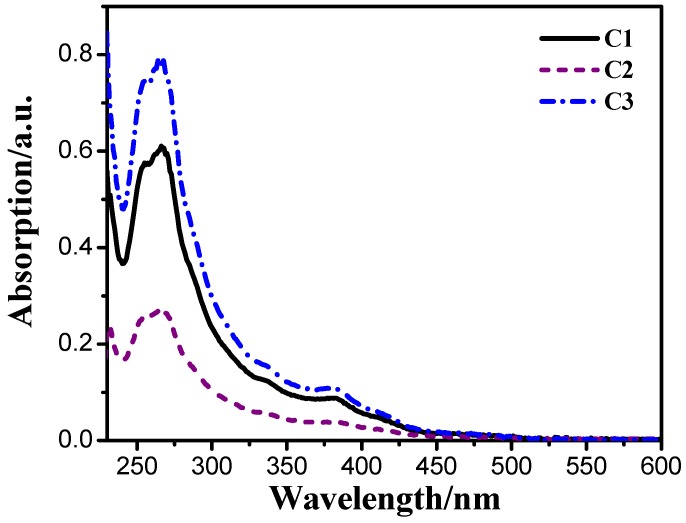
UV-Vis absorption spectra of the cationic iridium(III) complexes in CH_2_Cl_2_ at 1.0 × 10^−^^5^ mol·L^−1^ at room temperature.

The UV-visible absorption spectra of the iridium(III) complexes in CH_2_Cl_2_ solution of 1.0 × 10^−5^ mol·L^−1^ at room temperature are shown in Figure 2. The intense absorption bands in the ultra-violet region between 230 nm and 350 nm are ascribed to the spin-allowed ^1^π–π* transition of the ligands. The weak absorption band from 350 nm extending to the visible region are overlapping absorption of ^1^MLCT (metal-ligand charge-transfer), ^1^LLCT (ligand-to-ligand charge-transfer), ^3^MLCT, ^3^LLCT and ligand-centered (LC) ^3^π–π* transitions [20,29]. The absorption of spin-forbidden ^3^MLCT, ^3^LLCT and ^3^LCπ–π* mixing with higher-lying ^1^MLCT transitions exhibiting largish intensity is caused by the strong spin-orbit coupling endowed by the heavy iridium(III) atom [30,31]. Since all the absorption spectra of the complexes are caused by the same organic iridium(III) complex cation [Ir(ppy)_2_(phen)]^+^, the absorption spectra very much resemble one another, all of them have the same maximum absorption wavelength peaked at 267 nm. However, the absorption intensities of them at same wavelengths are different which means the absorption is affected by different anions of PF_6_^−^, SiF_6_^2−^ and TiF_6_^2−^. The complex C3 with TiF_6_^2−^ exhibits the maximum absorption intensity and that of the complex C2 with SiF_6_^2−^ is the minimum. 

### 3.2. Photoluminescent Properties

**Figure 3:**
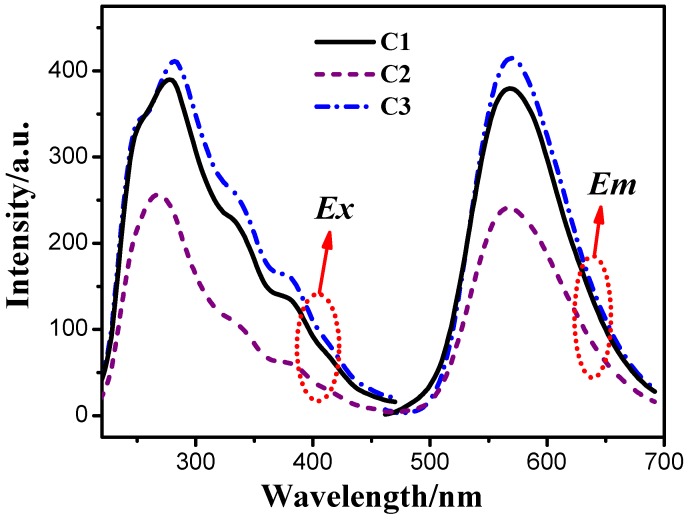
Excitation (*E*_x_, λ_em_ = 580 nm) and emission (*E*_m_, λ_ex_ = 342 nm) spectra of the cationic iridium(III) complexes in CH_2_Cl_2_ at 1.0 × 10^−^^5^ mol·L^−1^ at room temperature.

The excitation and emission spectra of the cationic iridium(III) complexes in CH_2_Cl_2_ solutions at 1.0 × 10^−5^ mol·L^−1^ at room temperature are shown in Figure 3. In addition, due to the same organic iridium(III) complex cation [Ir(ppy)_2_(phen)]^+^, three cationic iridium(III) complexes exhibit similar excitation and emission spectra. For complexes C1, C2 and C3, the maximum excitation wavelengths are 278 nm, 267 nm and 282 nm respectively, the maximum emission wavelengths are all 568nm. In general, for mixed-ligand cationic iridium(III) complexes, usually three excited states usually contribute to the observed light emission, those are ^3^LCπ–π*, ^3^MLCT and ^3^LLCT [32]. All complexes exhibit broad and almost featureless emission spectra, which demonstrated that the emissive excited states have predominantly ^3^LCπ–π* characters other than ^3^MLCT or ^3^LLCT [33]. 

**Figure 4:**
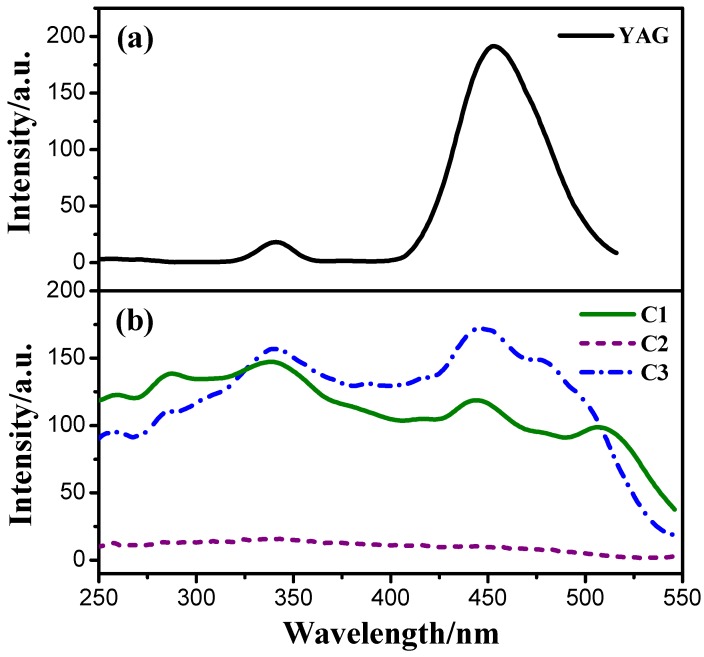
Excitation spectra (λ_em_ = 565 nm) of YAG (**a**) and the cationic iridium(III) complexes (**b**) powders.

**Figure 5:**
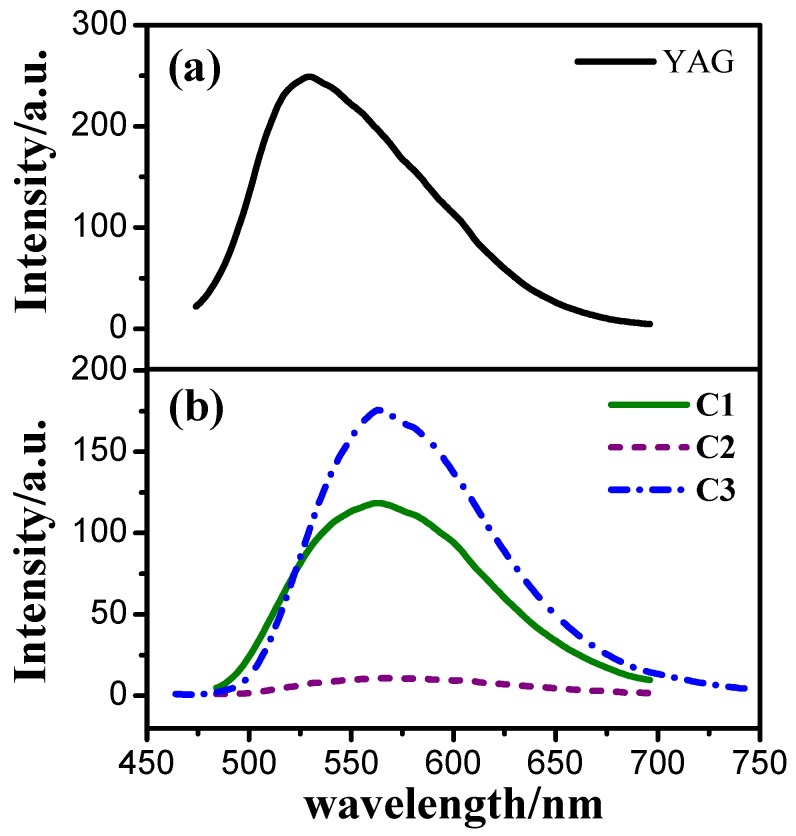
Emission spectra (λ_ex_ = 444 nm) of YAG (**a**) and the cationic iridium(III) complexes (**b**) powders.

Figure 4 and Figure 5 are excitation and emission spectra of the solid powders of YAG and three cationic iridium(III) complexes. As shown in Figure 4, at emission wavelength of 565 nm, the complexes all exhibit similar broad excitation bands from 250 nm to 550 nm, and all of them have two peaks with the maximum excitation wavelengths around 337 nm and 444 nm respectively. The YAG has a main peak at 400–515 nm with the maximum excitation wavelengths of 460 nm. In other words, all of the above-mentioned phosphors can be well excited by 460 nm emitting blue GaN chip and white light can be obtained by combining light from the chip and from one of the phosphors. However, as shown in Figure 5, the emission bands of YAG and cationic iridium(III) complexes are different. The emission of YAG mainly contains greenish yellow light, so its combination with 460 nm emitting blue GaN chip will obtain cool white light, on the contrary, the emission of the cationic iridium(III) complexes covers yellow light and part of orange red light, which can be coated on 460 nm emitting blue GaN chip for obtaining warm white light. From the excitation and emission spectra, another piece of information can be obtained, that the emission intensity of cationic iridium(III) complex C3 with TiF_6_^2−^ is higher than that of the others, likely due to its higher excitation light absorption. The order of emission intensities is C3 > C1 > C2, which means that different anions in the complexes will affect their emission intensities.

### 3.3. Thermal Stability

**Figure 6:**
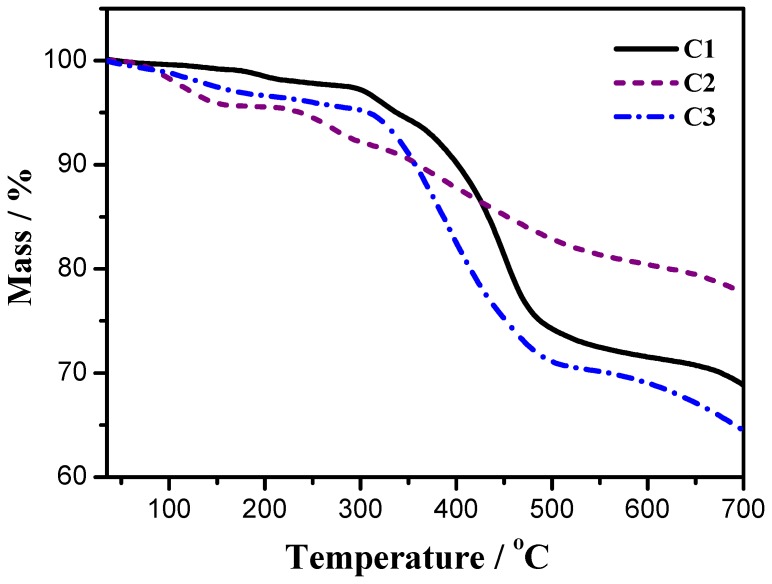
Thermogravimetric curves of the cationic iridium(III) complexes.

High thermal stability is an essential requirement for WLEDs, since WLEDs are fabricated and work usually at a temperature even near (but not exceeding) 150 °C [34]. The thermal properties of three cationic iridium(III) complexes are characterized by thermogravimetry (TG), and shown in Figure 6. At low temperature, approximately from room temperature to 220 °C for C1, from room temperature to 155 °C for C2, and from room temperature to 185 °C for C3 respectively, every complex has a little mass loss of adsorptive water and organic solvent residues, about 2.0% for C1, 4.0% for C2 and 3.0% for C3 respectively. Due to the easily degradable property of SiF_6_^2−^ (SiF_6_^2−^→ SiF_4_↑ + 2F^−^) [35], there is an obvious mass loss process between 200 °C and 300 °C (with a point of inflection at about 230 °C) on the TG curve of C2; however, C1 and C3 with stable anions of PF_6_^−^ and TiF_6_^2−^ do not show similar mass loss. At relatively high temperature, above 295 °C for C1, 345 °C for C2, and 315 °C for C3 respectively, every complex shows a big mass loss caused by the loss of neutral auxiliary ligands (1,10-phenanthroline) [22,36]. The results of thermal and optical properties suggest that complex C1 and complex C3 are suitable to be used in LEDs but complex C2 is unsuitable.

### 3.4. Fabrication and Performance of WLEDs

**Figure 7:**
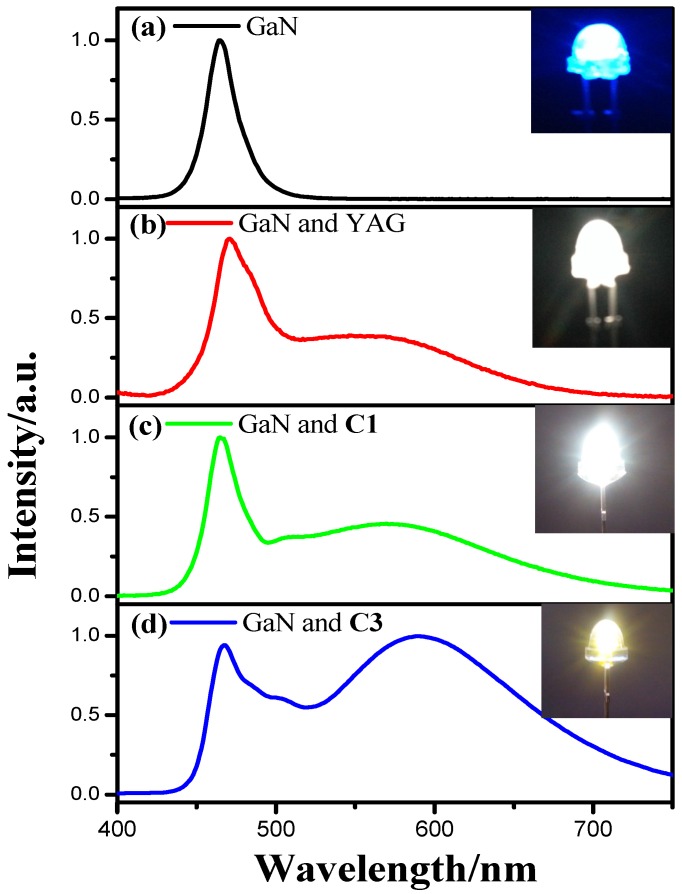
EL spectra of several LEDs at 20 mA forward bias: (**a**) The original blue GaN chip without phosphor (**b**) Blue GaN chip and YAG as phosphor (**c**) Blue GaN chip and complex C1 as phosphor (**d**) Blue GaN chip and complex C3 as phosphor.

In order to investigate the potential application of these cationic iridium(III) complexes, a series of WLEDs were fabricated by coating these complexes (doped in epoxy resin at mass ratio of 1:30) on the 460 nm emitting blue GaN chips. The electroluminescent (EL) spectra of these LEDs devices are shown in Figure 7. Figure 7a illustrates the EL spectrum of single LED chip with the strongest emission peaked at ~460 nm. Figure 7b is the EL spectrum of the WLED using the mixture of YAG and epoxy resin (the ratio of mass is 1:3) under 20 mA current excitation. The broad band in blue region is due to the emission of GaN chip, and the greenish yellow emission is due to the emission of YAG. The performance of this WLED is listed in Table 1. The WLED based on YAG exhibits high *T_c_* (7338 K) and low *R*_a_ (74.7). Figure 7c,d presents the EL spectra of the WLEDs based on the mixture of the cationic iridium(III) complexes and epoxy resin (the ratio of mass is 1:30). Some differences can be found in the EL spectra of these WLEDs from Figure 7. Firstly, the yellow emission part in spectrum of WLED with complex C3 shows obvious red-shift compared with that of YAG. Besides, the ration of blue emission and yellow emission in Figure 7d is smaller than that in Figure 7b. These results indicate that the WLED fabricated with complex C3 share better performance than that with YAG. The related parameters of these WLEDs are also listed in Table 1. Among these WLEDs based on iridium(III) complexes, the WLED fabricated with C3 shows the strongest white light, and shows lower *T_c_* (3717 K) and higher *R*_a_ (82.6) compared with those of WLEDs based on YAG and C1.Moreover, a little of complex C3 can share intense yellow emission excited by the emission of GaN chip, compared with YAG. Hence the complex C3 maybe find application in WLEDs. 

**Table 1:** Performances of LEDs under 20 mA current excitation.

LED	Mass ratio of Phosphor and Epoxy Resin	*T*_c_** (K)	*R*_a_	Luminous Efficiency (Lm/W)	CIE (*x, y*)
only blue GaN chip	‒	100000	49.5	12.91	(0.13, 0.06)
YAG and blue GaN chip	1:3	7338	74.7	14.61	(0.29, 0.35)
C1 and blue GaN chip	1:30	5912	82.0	10.61	(0.32, 0.35)
C3 and blue GaN chip	1:30	3717	82.6	11.41	(0.40, 0.40)

## 4. Conclusions

Three cationic iridium(III) complexes [Ir(ppy)_2_(phen)][PF_6_] (C1), [Ir(ppy)_2_(phen)]_2_SiF_6_ (C2) and [Ir(ppy)_2_(phen)]_2_TiF_6_ (C3) with different anions were synthesized. Complex C1 and C3 exhibit good optical properties and high thermal stability; however, these properties of complex C2 are poor, probably due to the easily degradable property and high water adsorption of SiF_6_^2−^. Complex C1 and C3 exhibit intense and broad greenish-yellow emission with broad excitation bands in blue region. The WLEDs fabricated using complex C1 and C3 as luminescence conversion materials show good optical performances that are better than those of the widely used yellow phosphor YAG; therefore, these two complexes (especially complex C3) are considered to be good candidates for WLEDs.

## Supplementary Materials

Supplementary materials can be accessed at: http://www.mdpi.com/1996-1944/8/9/6105/s1.
