# Supplementary material for: The Photoluminescent Properties of New Cationic Iridium(III) Complexes Using Different Anions and Their Applications in White Light-Emitting Diodes
Source: Materials (Basel). 2015 Sep 14;8(9):6105–16. doi: 10.3390/ma8095296 (PMC5512903; doi:10.3390/ma8095296)
Supplement: Supplementary file 1 [file materials-08-05296-s001.pdf]

Supplementary Materials

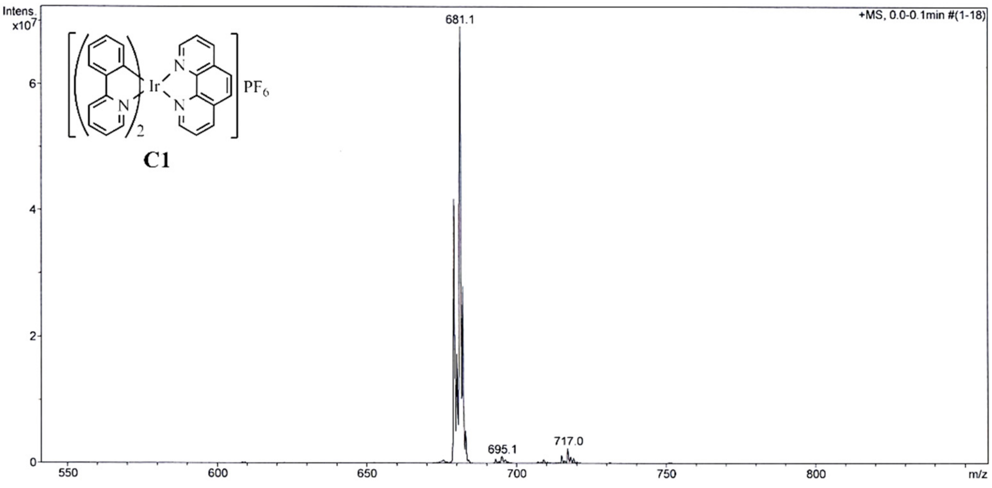

(**a**)

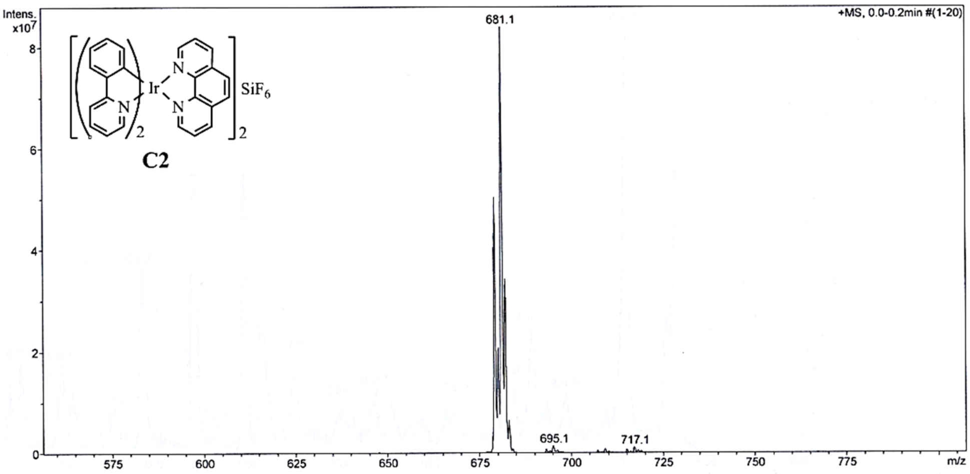

(**b**)

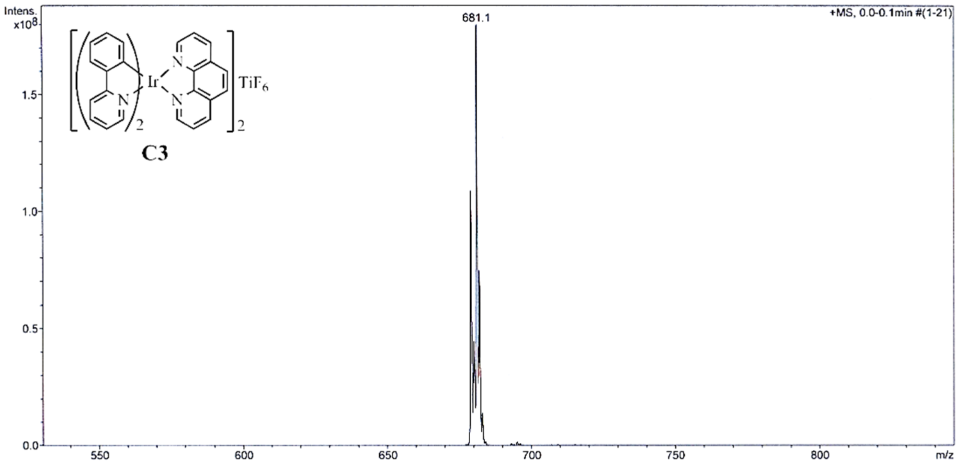

(**c**)

Figure S1. Mass spectra of (**a**) $[Ir(ppy)_2(phen)][PF_6]$ (C1); (**b**) $[Ir(ppy)_2(phen)]_2SiF_6$ (C2); and (**c**) $[Ir(ppy)_2(phen)]_2TiF_6$ (C3).
